# Are the senses enough for sense? Early high-level feedback shapes our comprehension of multisensory objects

**DOI:** 10.3389/fnint.2012.00082

**Published:** 2012-09-26

**Authors:** Lorina Naci, Kirsten I. Taylor, Rhodri Cusack, Lorraine K. Tyler

**Affiliations:** ^1^Department of Psychology, The Brain and Mind Institute, Western UniversityLondon, ON, Canada; ^2^Department of Experimental Psychology, Centre for Speech and Language, University of CambridgeCambridge, UK; ^3^Memory Clinic-Neuropsychology Center, University Hospital BaselBasel, Switzerland; ^4^Medical Research Council Cognition and Brain Sciences UnitCambridge, UK

**Keywords:** familiar, multisensory, integration, object, semantic, top–down, feedback

## Abstract

A key question in cognitive neuroscience is how the brain combines low-level features processed in remote sensory cortices to represent *meaningful* multisensory objects in our everyday environment. Models of visual object processing typically assume a feedforward cascade through the hierarchically organized ventral stream. We contrasted this feedforward view with an alternate hypothesis in which object processing is viewed as an interactive, feedforward and feedback process. We found that higher-order regions in anterior temporal (AT) and inferior prefrontal cortex (IPC) performed audio-visual (AV) integration 100 ms earlier than a sensory-driven region in the posterior occipital (pO) cortex, and were modulated by semantic variables (congruency), from as early as 50–100 ms. We propose that the brain represents familiar and complex multisensory objects through early interactivity between higher-order and sensory-driven regions. This interactivity may underpin the enhanced behavioral performance reported for semantically congruent AV objects.

## Introduction

To recognize a familiar object in our everyday environment (e.g., an animal or a tool), the brain effortlessly integrates inputs from different sensory modalities into a coherent meaningful representation. While multisensory integration responses have been consistently reported at numerous sites across the cortex, a key, unresolved question in cognitive neuroscience concerns the temporal mechanism that combines multisensory integration responses to familiar object features (e.g., the visual percept and roar of a lion) that occur across the brain into an object representation. fMRI studies have consistently reported audio-visual (AV) integration responses to complex stimuli in both auditory (A) and visual (V) sensory regions, and in higher-order anterior ventral regions. For example, regions in primary and association auditory and visual cortices (Calvert et al., 1999), traditionally thought to be sensory-specific, show AV integration responses during multisensory speech perception and object processing. AV responses to meaningful multisensory object stimuli have been reported in regions higher-up in the object processing hierarchy, including the lateral temporal (Beauchamp et al., 2004; Hein et al., 2007), anterior temporal (AT), and in particular the antero-medial temporal cortex (AMTC) (Taylor et al., 2006), prefrontal cortex (Laurienti et al., 2003), and inferior prefrontal cortex (IPC) (Hein et al., 2007). It remains unclear when AV integration responses in higher-order and sensory-driven regions interact, and whether integration responses from anterior ventral regions feed back to affect integration responses in posterior occipital (pO) regions from early stages of multisensory object processing.

Research on *visual* object recognition provides two models that may explain how multisensory integration responses in sensory-driven and higher-order regions are combined during the early stages of multisensory object processing. The traditional, feedforward model (Riesenhuber and Poggio, 2000) claims that object recognition is achieved through a feedforward, bottom–up processing cascade from sensory-driven to higher-order regions, where the evaluation of the meaning of an object is carried out at the final stages of object processing. However, an important architectural aspect of the visual system, i.e., the anatomical back projections between almost all ventral stream sites (Felleman and Van Essen, 1991), appears incompatible with a strictly feedforward view. Indeed, studies on visual object recognition (Barceló et al., 2000; Bar et al., 2006) instead support an interactive feedforward and feedback (top–down) model of object recognition, whereby processes in higher-order regions influence those in sensory-driven regions from the earliest stages, prior to recognition, and before a fine-grained meaningful representation has been achieved (Lamme and Roelfsema, 2000; Bullier, 2001; Bar et al., 2006; Clarke et al., 2011).

Nevertheless, models of the temporal dynamics of multisensory integration have been largely influenced by the feedforward view of visual object recognition. According to feedforward accounts of multisensory integration, auditory (A) and visual (V) inputs are analyzed within separate, hierarchically structured sensory processing streams, whose outputs finally become integrated in higher-order, multisensory sites (Felleman and Van Essen, 1991; Stein and Meredith, 1993). One such site is the AMTC (Simmons and Barsalou, 2003), where polymodal neurons bind inputs from the different sensory modalities together (Murray and Richmond, 2001). Thus, this view assumes that multisensory integration responses in higher-order regions occur at later stages, i.e., after extensive processing has taken place in sensory-specific streams, and does not allow for early interactions between higher-order and sensory-driven integration responses (Felleman and Van Essen, 1991; Calvert, 2001).

The strict independence of unisensory processing has been called into question by reports of rapid AV integration responses (i.e., below 100 ms) in auditory (Foxe et al., 2000) and visual (Giard and Peronnet, 1999) cortex. Based in part on findings of direct connections between visual areas V1 and V2, and the core belt and parabelt auditory areas in the macaque monkey (Falchier et al., 2002), these early effects have been attributed to direct interactions between sensory cortices, independent of top–down triggers (Foxe and Schroeder, 2005). Thus, the feedforward, hierarchical account has been modified to allow for early interactions between unisensory cortices. The multisensory responses from the sensory-driven regions are proposed to feed forward to higher-order regions, where recognition is accomplished.

An alternative, interactive account of multisensory integration across the brain would claim that early AV integration responses, which may result from direct interactions between the sensory cortices, are modulated in a top–down fashion by on-going AV integration responses in higher-order regions, and that these interactions occur before multisensory object recognition. To determine whether AV integration involves early top–down feedback, as suggested for visual object processing (Barceló et al., 2000; Lamme and Roelfsema, 2000; Miyashita and Hayashi, 2000; Bar et al., 2006), and what its role might be, we exploited the temporal sensitivity of EEG recordings and investigated the time-course of AV integration responses in sensory-driven and higher-order regions.

Two candidate regions for early top–down feedback exist within the ventral object processing system, and both appear critical for processing meaningful aspects of AV object stimuli: the ventral portion of the orbitofrontal cortex (OFC) located within the IPC region, and the AMTC located in the AT region. The AMTC and OFC are among the most heteromodal cortical regions, receiving afferents from all sensory modalities (Kringelbach, 2005). Both show multisensory responses in monkeys (Murray and Richmond, 2001; Romanski, 2007) and humans (Taylor et al., 2006). Importantly, activation in both the AT and IPC regions is modulated by high-level object information in monkeys (Sugase et al., 1999; Freedman et al., 2001), and by semantic variables in humans (Moss et al., 2005; Hein et al., 2007). Within the AMTC, the perirhinal cortex, located at the culmination of the occipito-temporal portion of the ventral object processing stream, is specifically involved in differentiating objects that share many properties and are therefore ambiguous (Moss et al., 2005; Barense et al., 2007; Clarke et al., 2011). The IPC is involved in processing visual object identity (Ranganath, 2006) and is thus hypothesized to represent the prefrontal extent of the object processing stream (Ungerleider and Haxby, 1994). Within the IPC, the ventral OFC plays a multifaceted role in object processing. This includes context-dependent semantic processing of objects to determine their behavioral meaning (Miller and Cohen, 2001), and context-independent processing of low visual spatial frequencies to determine the form of visual objects, starting from as early as 150 ms (Bar et al., 2006). Although the time-course of human AMTC involvement in object processing has not been investigated, findings of its direct connections with the pO cortex via the inferior longitudinal fasciculus (Catani et al., 2002) and strong bilateral connections with the OFC (Kringelbach, 2005) suggest that it may play an early top–down role in AV object processing.

To investigate the spatiotemporal profile of AV integration responses in a set of theoretically and empirically motivated regions of interest (ROIs), we performed source analyses of EEG data. Two higher-order regions, one in AT and one in IPC, were defined as the sites onto which the activity from our medial ROIs, the AMTC and OFC, respectively, would most likely be localized by the distributed source modeling method. The more lateral AT (Mummery et al., 2000) and IPC (Wagner et al., 2001) regions have inherent semantic processing capacities. In addition, we defined a sensory-driven/auditory region in the lateral superior temporal (ST) cortex, and a sensory-driven/visual region in the lateral pO cortex.

We used pairs of A, V, and AV stimuli (i.e., two image parts, two sounds, or a sound and an image) to represent familiar objects (e.g., animals and tools), and manipulated object meaning via the variable of semantic congruency. EEG data were recorded while participants made semantic congruency decisions in each unisensory (A, V) and cross-sensory (AV) trial. Stimuli in congruent trials represented the same object (e.g., a complete picture of a lion and the sound “roar”), whereas stimuli in incongruent trials represented different objects (e.g., a complete telephone picture and the sound “woosh”). By measuring responses to stimuli that could be either meaningfully integrated (congruent) or not (incongruent), we were able to evaluate each region's response to the semantic relationship between A and V stimuli, over time.

We asked two related questions. First, we tested whether the AT and IPC regions are involved in *early* stages of familiar AV object processing (<150 ms), i.e., prior to the onset of the EEG components correlated with object recognition (Johnson and Olshausen, 2003). Second, we tested whether early AV responses in AT and IPC reflected semantic processing. We predicted that semantic congruency would modulate AV integration responses in the AT and IPC regions, based on reports of AV semantic congruency effects in the AMTC (Taylor et al., 2006) and IPC (Hein et al., 2007). If the emergence of a familiar object representation is underpinned by early top–down feedback, then semantic congruency will modulate early AV integration responses in these regions.

## Materials and methods

### Participants

Eighteen healthy volunteers (age-range 18–40 years; 13 males) with normal or corrected-to-normal vision participated. Participants had no history of neurological disorders and did not take any psychotropic or drowsiness-inducing medication. All were right-handed, as determined by the Edinburgh Handedness Inventory (Oldfield, 1971), and gave informed consent. The study was approved by the Cambridge Psychology Research Ethics Committee.

### Materials

**Figure 1:**
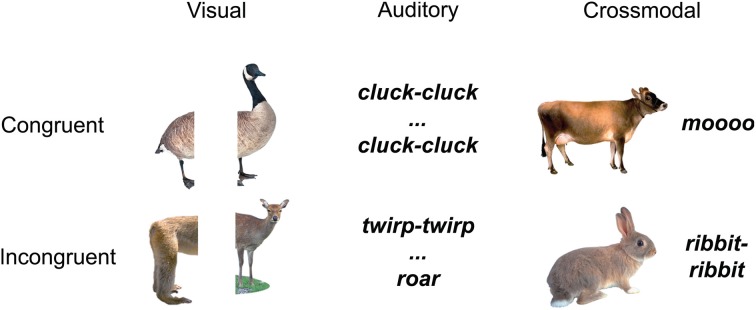
**Example stimuli belonging to the living things domain**.

The stimuli were naturalistic color photographs (Figure 1) and environmental sounds of living and non-living things (e.g., animals and tools/appliances). *All* conditions (auditory baseline, visual baseline, crossmodal condition) used concepts from the *same* living and non-living categories, i.e., animals and tools/appliances. Each category had an equal number of living and non-living things, and within each domain, an equal number of congruent and incongruent stimuli.

One of the unique contributions of this experiment, as compared to other experiments investigating cross-modal integration, is that here we look at the effects of *integration* that are unique to *cross-modal* (combined auditory and visual) stimuli, as compared to effects of integration of *unisensory* (auditory or visual) stimuli. In order to be able to investigate unisensory and cross-modal integraton within the same paradigm, we created conditions where the integration of the stimuli could take place in each sensory modality, as well as across modalities, independently of one another. To avoid any priming effects across conditions, we created stimuli that were unique within each condition.

Another motivation for the stimuli selection was to keep them the same as those used in a previous fMRI experiment (Taylor et al., 2006, *PNAS*). This would enable us to compare the effects of cross-modal integration across neuroimaging modalities (see “Discussion” section).

The unisensory visual (V) trials (*n* = 100) consisted of two picture halves, the unisensory auditory (A) trials (*n* = 100) consisted of two sound parts, and the AV trials (*n* = 100) consisted of whole pictures and whole environmental sounds. All 100 AV and all 200 unimodal stimuli were unique. In half of the trials of each condition the two stimulus parts were congruent, and in the other half of the trials, in each condition, they were incongruent. Specifically, for the congruent unisensory visual (V) trials we used two halves of the same objects (i.e., from the same image), whereas for the incongruent visual trials, we used two halves from pictures of different objects (within-domain) (e.g., congruent V: left half of a cat picture on left, right half of a cat picture on right; incongruent V: left part of a dog picture on left and right part of a cow picture on right; congruent A: the sound “jjj” followed by its other sound half “jjj”; incongruent A: the sound “moo” followed the part of another sound “clack”; congruent AV: a complete picture of a lion and the whole environmental sound “roar”; incongruent AV: a complete picture of a telephone and the whole environmental sound “woosh”). Within each congruency condition, half of the trials represented living and half non-living things.

Critically, continuity between stimuli pairs in the incongruent trials was addressed by making sure that incongruent trials presented stimuli (images/sounds) from the same semantic category (i.e., they were both animals, or tools, etc.). Thus, we were able to avoid confounding the effects of semantic congruency with effects due to semantic domain.

Images were presented on a grey background of a 21-inch computer monitor placed 45 cm in front of the participant, and with a screen resolution of 1024 × 768 pixels and refresh rate of 60 Hz. The sounds were matched for peak amplitude (−7.7 dB). They were truncated to have the same length (1185 ms) for all the AV trials. For the A trials, the environmental sounds were divided into two halves with length of 593 ms and 592 ms. We also included control trials consisting of pairs of visual noise picture halves (Vscrambled), pink noise filtered environmental sound halves (Anoise), and visual noise whole pictures with pink noise transformed environmental whole sounds (AVnoise and scrambled) (*n* = 52 in each condition), to control for the effects of low-level visual and auditory information processing on meaningful unisensory and multisensory object integration. In order to create the Anoise stimuli, the environmental sounds were transformed into pink noise by using the “generate noise” (pink) option in the Audacity software (http://audacity.sourceforge.net/). The noise filter was applied for the entire duration of the sound. The Vscrambled stimuli were created in Photoshop (Adobe Photoshop CS5, Version 12.0 × 64) by applying the “noise” filter to each image. This filter added 100% Gaussian distributed noise to the image.

### Task

Participants were presented with an environmental sound and a picture (e.g., the sound “roar” and a picture of a lion) in the AV condition, and two parts of a sound and two parts of a picture in the unimodal A and V conditions, respectively. Participants decided, for every trial, whether the two items were congruent or incongruent by pressing different response keys. This design allowed us to isolate the processes unique to meaningful integration of object features across sensory modalities, as different from integration *per se* and associated decision-making processes, by contrasting neural responses to the AV integration conditions with the sum of the responses due to unimodal (A + V) integration.

### Procedure

E-Prime (Psychology Software Tools) was used to present and control the timing of the stimuli, and to communicate with the data acquisition software (Net Station; Electrical Geodesics, Inc.). In the unimodal conditions, participants were presented with two halves of stimuli. In the visual condition, the two image parts were presented simultaneously, and in the auditory condition, the two parts of the auditory object/two sounds were presented sequentially, separated by 750 ms of silence. The V and AV trials were 1185 ms long, whereas the A trials were 1835 ms, including the silence. The stimuli were pseudo-randomly presented in fours blocks of 114 trials each. Within each block, the trial types were pseudo-randomized and the SOA jittered, between 1000 and 3200 ms. The order of block presentation was counterbalanced across subjects. Participants pressed a key to indicate whether the two stimuli were congruent or incongruent, and did not respond during the control trials. To avoid the motor response overlaying on the electrical activity due to integration processes, participants were instructed to not respond as soon they knew the answer, but, rather, to wait until the end of the trial before making a response. The resulting RT data were considered inadequate for analysis.

### Data acquisition and pre-processing

Continuous EEG was acquired from 128 scalp electrodes (impedances <50 kΩ), band pass filtered between 0.01 and 100 Hz and digitized at 250 Hz, using a Geodesic EEG System 250 (Electrical Geodesics, Inc.). The data were band-pass filtered offline with a 0.1–40 Hz forward filter to remove low frequency drifts as well as high frequency noise, including line noise. The continuous EEG was divided into epochs from −200 ms pre- to 800 ms post-stimulus presentation. Trials contaminated by blinks and horizontal eye movements were rejected off-line on the basis of vertical and horizontal electro-oculograms. In addition, exclusion criteria for amplitude >100 μV and gradient >70 μV were used to reject trials with excessive EMG and other noise transients. Participants with artifacts in more than 20% of the object trials were excluded from further analysis (*n* = 3), to ensure adequate power in the source localization analysis. Average referenced EEG data were submitted to ERP analyses and source modeling.

### ERP analysis

EEG epochs were sorted according to each condition and averaged for each subject to compute individual subject ERPs. Group averaged ERPs for each condition were calculated for display and analysis purposes. Consistent with previous studies, AV integration responses were defined as AV > (A + V). (When calculating the sum A + V, we used the second part of the auditory trial, as the unimodal auditory objects gradually unfolded in time and all the auditory stimulus information would be available during the second sound.) The stringent criterion of super-additivity [AV > (A + V)] was used to avoid false positives when measuring AV responses, or responses due to the concurrent processing and integration of A and V stimuli (Giard and Peronnet, 1999; Foxe et al., 2000; Molholm et al., 2002). The latency window and electrode sites for the visual N1 in the AV condition were defined based on the unisensory V condition, before assessing the effect of AV integration processes. The mean ERP values (N1 interval) were averaged across montages of electrodes from the left and right pO regions (four per hemisphere), and entered into repeated measures ANOVA with the factors *Hemisphere* (2), and *Condition* (2: AV, A + V).

### Distributed source modeling

To investigate the cortical generators that underlie AV integration, and in particular to reveal the time-course of their responses, Minimum-Norm Current Estimates (MNCEs) were calculated. L2 minimum norm was computed using Brain Electric Source Analysis software (BESA 5.1, MEGIS Software GmbH, Munich). The 128 electrode positions were transformed to head coordinates using the standard BESA 5.1 brain. An idealized four-shell ellipsoidal head model (Berg and Scherg, 1994) with a radius of 92.5 mm, and scalp, skull and CSF thickness of, respectively, 6 mm, 7 mm, and 1 mm were used to calculate the EEG forward solution, before the inverse solution was computed. BESA modeled the neural activity from medial and lateral sources by projecting it on the lateral surface of the cortex. In total, there were 1426 evenly distributed regional sources (713 per hemisphere), each consisting of three orthogonally oriented dipoles, which modeled the electrical activity across the cortex at each time sample (4 ms). To account for the contribution of deep sources, the L2 minimum norm was computed for a source configuration consisting of two layers of regional sources 10 and 30% below the cortical surface. Thus, for each location on the lateral surface of the cortex, the minimum norm was computed for two regional sources below it. The larger activity of the two sources was projected onto the lateral surface of the cortex. This source placement is a standard feature of the BESA software.

### ROI analyses

Based on the MNCEs, ROI waveforms (group and individual data) were extracted for four ROIs bilaterally, located on the pO, ST, AT, and IPC. The particular location of the AT and IPC ROIs on the lateral surface of the cortex was chosen to optimize the detection of the response from the medial sources of interest (AMTC and OFC). ROI waveforms were computed by averaging, at each time sample, the strength of sources within the boundaries of each ROI, defined by Brodmann (BA) areas in MRIcro (www.mricro.com) (pO: BA 17, 18; ST: BA 41, 42; IPC: BA 45, 47; AT: BA 38). For statistical comparisons, the data was averaged along empirically and theoretically latency regions, based on 100 ms or 50 ms time-intervals locked to stimulus presentation, thus avoiding biasing the statistical results (Vul et al., 2009). Within each condition (A, V, and AV), the ROI activity was investigated by entering averaged ROI responses (100 ms time-bin) into repeated measures ANOVAs with factors *Time*, *Hemisphere* and *ROI*. The Huynh-Feldt correction was applied to spherical-data. Planned paired *T*-tests or independent sample *T*-tests were used to explore significant effects of ANOVA, or test *a priori* hypotheses.

### Regional response analyses

In the A trials, the two parts of the auditory object/two sounds were presented sequentially, separated by 750 ms of silence. The first and second parts of the auditory object (separate sounds) were averaged and analyzed independently, as the unimodal auditory objects gradually unfolded in time, and the underlying neural processes were expected to differ. Specifically, in the context of the semantic congruency task, no integration could take place during the first sound. Separate repeated measures ANOVAs with factors *Hemisphere* (2), *ROI* (3), and *Time* (4) were run on responses from each sound. Significant effects were explored further with planned paired *T*-test comparisons of the responses in the ST to those in the AT/IPC regions. Different repeated measures ANOVA with factors *Hemisphere* (2), *ROI* (3), and *Time* (4) were performed separately for each set of Vscrambled, V, and AV trials. Significant effects were explored further with planned paired *T*-test comparisons of the responses in the pO to those in the AT/IPC regions. Regional responses were collapsed across hemispheres, to limit the number of comparisons.

### Analysis of AV integration

Similarly to the ERP analysis, the criterion of super-additivity [AV > (A + V)] was used to calculate AV integration responses in the source-localized data. As mentioned above, when calculating the sum A + V, we used the second part of the auditory trial. Fifty millisecond time-intervals were used when testing the difference between conditions [e. g., AV – (A + V)], to ensure adequate temporal resolution of subtle effects. The effect of semantic processing on AV integration responses was investigated across semantic congruency trials by comparing AV integration responses in congruent and incongruent trials [[AV congruent > (A + V) congruent] – [AV incongruent > (A + V) incongruent]]. Effects of semantic congruency were explored pre-150 ms, in two intervals (50–100 ms, 100–150 ms), determined by orthogonal analysis, with repeated measures ANOVA with factors *Hemisphere* (2), *ROI* (3), and *Time* (2 or 3). Significant effects of the ANOVAs were explored further by planned independent sample *T*-tests.

## Results

### ERP analysis

**Figure 2:**
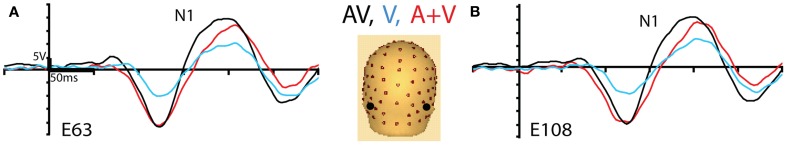
**Group averaged ERPs for AV trials (black trace), V trials (blue trace), and the sum of unisensory (A + V) trials (red trace), (A) at left and (B) right posterior occipital (pO) electrode sites, indicated in black on the 3D electrode layout**.

Initially, we tested for early AV integration responses on the scalp-based visual ERPs. We found an enhancement of the visual N1 component during AV trials compared to the sum of unisensory (A + V) trials, at pO sensors, in the latency-window 150–200 ms (Figure 2). A repeated measures ANOVA with the factors *Hemisphere* (2) and *Condition* (2) showed a significant main effect of *Condition* [*F*_(1, 14)_ = 23, *p* < 0.001], with the AV response significantly more negative-going than the sum of unisensory responses (A + V). This finding replicates earlier reports (Giard and Peronnet, 1999; Molholm et al., 2002; Molholm, 2004).

### Source modeling analysis

We then analyzed the source-localized unimodal (A/V) responses for proof of principle that the ST and pO regions were primarily driven by sensory processes, whereas the AT and IPC regions were driven by higher-order processes. Although sensory/bottom-up processes and higher-order/top–down processes may occur throughout the time-course of object processing, we expected the former to dominate early (0–200 ms), and the latter to dominate subsequent (200–400 ms) processing stages. Thus, we expected unimodal ST/pO responses to be stronger than AT/IPC responses between 0–200 ms, and the reverse to be true from 200–400 ms.

Initially, we tested regional responses during unisensory auditory integration. In this context, we refer to *auditory integration* as the process by which two sounds naturally merge into a longer, coherent auditory percept. For instance, each congruent auditory trial presented two halves of the same environmental sound (e.g., the sound “jjj” followed by its other sound half “jjj”). Therefore, in a congruent auditory trial, during the presentation of the second sound, integration occurred naturally, as the two sounds merged into one percept. By contrast, each incongruent auditory trial presented two halves of different sounds (e.g., the sound “moo” followed the part of another sound, e.g., “clack”). As a result, in an incongruent auditory trial, the two sounds clearly did not go together and did not form a coherent whole. At the end of the trial, the two sounds were still perceived as two separate items.

**Figure 3:**
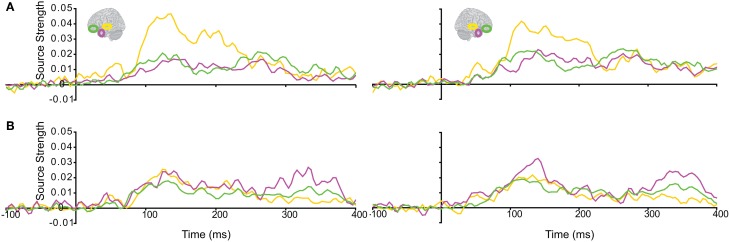
**Regional response waveforms averaged across participants during (A) the first sound, when integration could not yet occur, and (B) the second sound, when participants attempted to integrate the two sounds into a meaningful auditory object.** Left/right panel displays left/right hemisphere.

**Figure 4:**
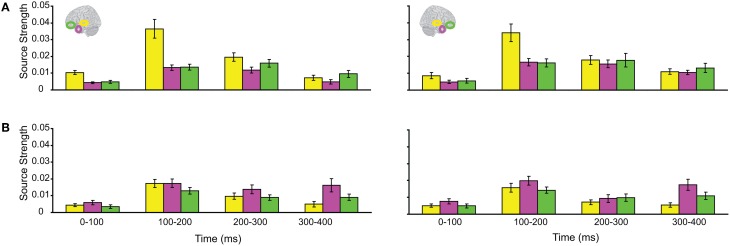
**Time-binned (100 ms) responses to auditory trials (same as Figure 3)**.

The responses during each sound were analyzed separately, as integration could not yet occur during the 1st sound. (We did not use the Anoise sounds in this analysis. The 1st sound served as a low-level baseline for the 2nd sound, when all the auditory information could be integrated into a familiar auditory object.) Figure 3 displays regional response waveforms for each sound. Figure 4 displays these responses time-binned (100 ms) for statistical analyses. For the 1st sound, repeated measures ANOVA with factors *Hemisphere* (2), *ROI* (3), and *Time* (4) showed (a) a significant effect of *ROI* and (b) a significant *ROI by Time* interaction. These were driven by large response fluctuations in the ST region compared to the relatively small changes in the AT/IPC responses over time [a: *F*_(2, 82)_ = 11.7; *p* < 0.001; b: *F*_(6, 84)_ = 13; *p* < 0.001]. Paired *T*-tests of regional responses showed (a) ST > AT and (b) ST > IPC, from 0–100 ms [a: *t*_(14)_ = 4.2; *p* = 0.001; b: *t*_(14)_ = 2.8; *p* < 0.05] (Figure 4A). Regional dominance was reversed during the 2nd sound. Repeated measures ANOVA with factors *Hemisphere* (2), *ROI* (3), and *Time* (4) showed (a) a significant effect of *ROI* and (b) a significant *ROI by Time* interaction. These were driven both by the decrease of ST and increase of AT/IPC responses over time [a: *F*_(2, 82)_ = 10.3; *p* < 0.001; b: *F*_(6, 84)_ = 5.2; *p* < 0.001]. Paired *T*-tests of regional responses showed (a) AT > ST, and (b) IPC > ST, from 300 to 400 ms [a: *t*_(14)_ = 4.3; *p* = 0.001; b: *t*_(14)_ = 3.2; *p* < 0.01] (Figure 4B). In summary, we found dominance of ST over AT and IPC responses during the 1st sound, and the reverse effect during the later stages of the 2nd sound processing (time >200 ms), when integration between the two sounds could take place. This suggested that the ST region had greater involvement than higher-order regions in sensory processes. By contrast, the AT and IPC regions had greater involvement than sensory regions in the integration of the two sounds into a familiar auditory object.

**Figure 5:**
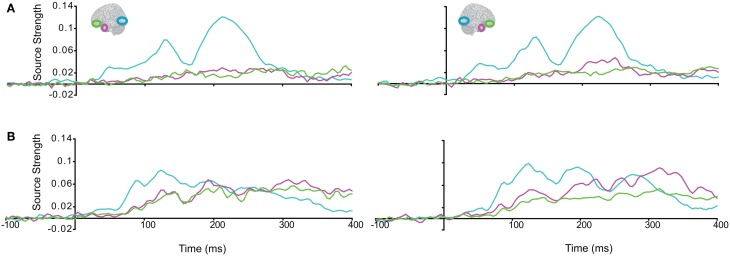
**Regional response waveforms to visual trials, averaged across participants, during (A) the Vscrambled trials, presenting phase-scrambled visual images, and (B) the Vobject trials, presenting two object (s) parts**.

**Figure 6:**
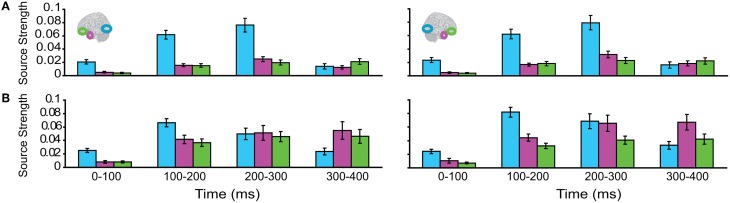
**Time binned (100 ms) responses to visual trials (same as Figure 5)**.

**Figure 7:**
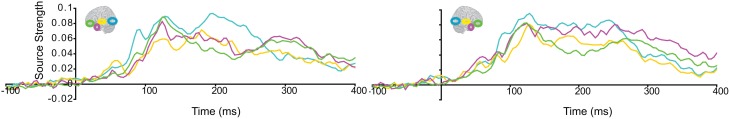
**Regional response waveforms to intact audio-visual object trials, averaged across participants**.

**Figure 8:**
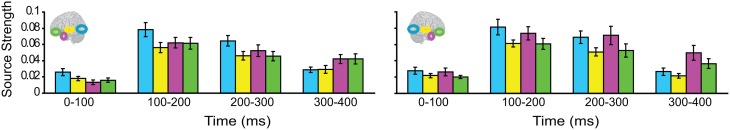
**Time binned (100 ms) responses to audio-visual trials (same as Figure 7)**.

Subsequently, we tested regional responses during unisensory visual processing. We compared responses to phase-scrambled visual stimuli, with those to intact visual objects. Figure 5 displays regional response waveforms for scrambled objects (Figure 5A) and intact objects (Figure 5B). Figure 6 displays these responses time-binned (100 ms) for statistical analyses. For scrambled objects, repeated measures ANOVA with factors *Hemisphere* (2), *ROI* (3), and *Time* (4) showed (a) a significant effect of *ROI* and (b) a significant *ROI by Time* interaction. These were driven by the peaking and subsiding pattern of pO responses versus the relatively small changes in the AT/IPC responses over time [a: *F*_(2, 82)_ = 28; *p* < 0.001; b: *F*_(6, 84)_ = 28.2; *p* < 0.001]. Paired *t*-tests of ROI strength showed (a) pO > AT and (b) pO > IPC, from 0–100 ms [a: *t*_(14)_ = 5; *p* < 0.001; b: *t*_(14)_ = 5.4; *p* < 0.001] (Figure 6A); similarly, from 100–200 ms, (a) pO > AT and (b) pO > IPC [a: *t*_(14)_ = 5.3; *p* < 0.001; b: *t*_(14)_ = 4.7; *p* < 0.001]; also, from 200 to 300 ms, (a) pO > AT and (b) pO > IPC [a: *t*_(14)_ = 3.3; *p* < 0.005; b: *t*_(14)_ = 3.5; *p* < 0.005]. By contrast, for visual objects, repeated measures ANOVA with the factors *Hemisphere* (2), *ROI* (3), and *Time* (4) showed a significant *ROI by Time* interaction. This was driven by interleaved (peaking and subsiding) responses in the pO and AT/IPC regions over time [*F*_(6, 84)_ = 13.3; *p* < 0.001]. Paired *t*-tests of ROI strength showed that, from 0–100 ms, (a) pO > AT, and (b) pO > IPC, and from 300–400 ms, (c) AT > pO [a: *t*_(14)_ = 4.6; *p* < 0.001; b: *t*_(14)_ = 4.9; *p* < 0.001; c: *t*_(14)_ = 2.5; *p* < 0.05] (Figure 6B). In summary, during scrambled object trials, we found dominance of pO over AT/IPC responses. By contrast, during intact object trials, we found dominance of pO over AT/IPC responses, only at early stages (0–200 ms). This pattern reversed for the AT region in the latter stages (300–400 ms) of object processing. These results suggested that (similarly as for unisensory auditory trials) during unisensory visual object trials, the AT and IPC regions had greater involvement than sensory regions, when stimuli could be integrated into familiar objects (intact objects), as compared to when stimuli could not be integrated (scrambled objects). Responses during AV trials showed a similar bilateral pattern of decreasing responses in pO, accompanied by increasing responses in the AT and IPC regions, during 0–400 ms (Figure 7). Repeated Measures ANOVA with factors *Hemisphere* (2), *ROI* (4), and *Time* (4) showed a significant *ROI by Time* interaction. This was driven by the interleaved pattern (peaking and subsiding) of responses in the pO and AT/IPC regions [*F*_(9, 126)_ = 5; *p* < 0.001] (Figure 8). We analysed the AV trials further, to test for responses unique to AV integration [AV > (A + V)].

### Multisensory integration

**Figure 9:**
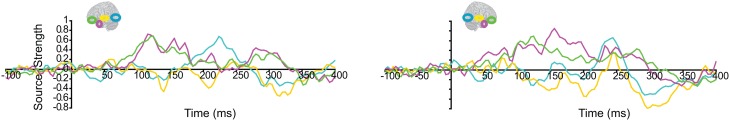
**Regional waveforms showing audio-visual integration responses [AV – (A + V)], averaged across participants, in the left and right hemispheres**.

**Figure 10:**
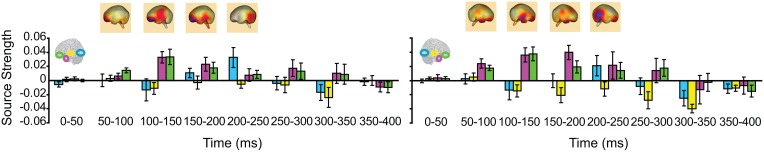
**Time-binned (50 ms) audio-visual integration responses (same as in Figure 9).** The top panel shows L2 minimum norm images from the whole-brain, which represent unconstrained localization of the group-averaged responses, for each respective time-interval. Goodness of fit = 96.3%.

We then turned to our main question of whether the AT and IPC regions were involved early (pre-150 ms) in AV integration. All trials (collapsed across semantic domains and semantic congruency categories), except for the AVscrambled (noise), were used for this analysis. (AV scrambled trials were not needed, as the variable of semantic congruency was used to investigate the effect of *semantic processing* during AV integration). Figure 9 displays regional waveforms for AV integration responses. Figure 10 displays the time-binned (50 ms) responses used in the statistical analyses. The ST region was not included in these analyses, as it did not exhibit AV integration responses (i.e., AV < A + V) (Figures 9, 10). Repeated measures ANOVA on the AV integration responses with factors *hemisphere*, *ROI* (pO, AT, and IPC), and *time* (50 ms steps from 50 to 400 ms) showed a significant *ROI by time* interaction, which was driven by the sequential pattern of AV responses in the AT/IPC and pO regions [*F*_(12, 168)_ = 3.2; *p* < 0.005]. Planned comparisons of AV responses were performed for the anterior regions in the time-intervals 50–100 ms and 100–150 ms. We found early, bilateral AV integration responses in (a) the left AT, from 100 to 150 ms, (b) the right AT, from 50 to 100 ms, (c) the left IPC, from 50 to 100 ms and (d) the right IPC, from 50 to 100 ms ROIs [a: *t*_(14)_ = 4.03; *p* = 0.001; b: *t*_(14)_ = 3.6; *p* < 0.005; c: *t*_(14)_ = 4.25; *p* = 0.001; d: *t*_(14)_ = 4.53; *p* < 0.001] (Figure 10). The early AT and IPC responses cannot be explained by increased eye movements during the AV trials, as trials contaminated by blinks and eye movements were removed prior to localization analysis. We also found significant AV integration responses in the left pO region, peaking at 200–250 ms [*t*_(14)_ = 2.3; *p* < 0.05]. These regional results were corroborated by the whole-brain, unconstrained localization (L2 minimum norm) of the integration responses across the entire lateral cortical surface (top panel in Figure 10). In summary, AV integration responses in the AT and IPC preceded by 100 ms those in the pO region (200–250 ms), suggesting early top–down feedback from these regions during AV integration.

### Semantic multisensory integration

**Figure 11:**
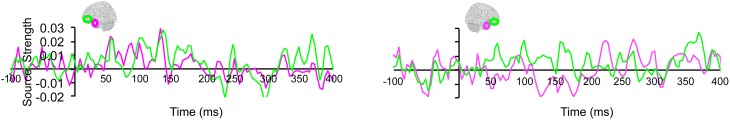
**Regional waveforms showing the difference between integration responses in congruent and incongruent AV object trials, averaged across participants, in the left and right hemispheres**.

**Figure 12:**
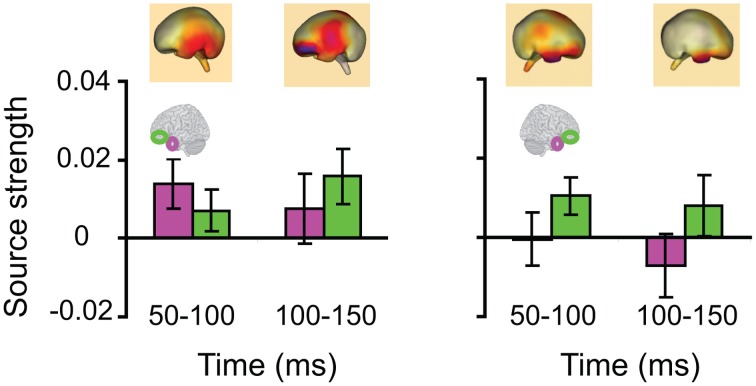
**Time-binned (50 ms) *semantic* audio-visual integration responses (same as in Figure 11), averaged across participants, at 50–100 ms and 100–150 ms.** The top panel shows L2 minimum norm images from the whole-brain, which represent unconstrained localization of the group-averaged responses, for each respective time-interval. Goodness of fit = 91.8%.

Lastly, we tested whether the *early* integration responses in AT and IPC regions was modulated by semantic integration. We compared integration responses in congruent and incongruent trials, during two intervals (50–100 ms, 100–150 ms), determined from the previous orthogonal analysis (Figures 11, 12). Repeated measures ANOVA with factors *hemisphere*, *ROI* (AT, IPC) and *time* (50–100 ms; 100–150 ms) showed a significant main effect of *hemisphere* reflecting stronger responses in the left hemisphere [*F*_(1, 14)_ = 4.23; *p* = 0.05]. Planned post-hoc comparisons revealed significantly stronger AV integration responses for congruent than incongruent AV stimuli (a) in the left AT, between 50 and 100 ms, and (b) in the left IPC, between 100 and 150 ms [a: *t*_(14)_ = 2.2; *p* < 0.05; b: *t*_(14)_ = 2.3; *p* < 0.05]. These regional results were corroborated by the whole-brain, unconstrained localization (L2 minimum norm) of the semantic effect across the entire lateral cortical surface (top panel in Figure 12). This result suggested that AT and IPC regions play an early role in the semantic integration of auditory and visual object features, from as early as 50–100 ms. No early effects of semantic domain were observed.

## Discussion

We tested whether the AT and IPC play an early (<150 ms) role in the semantic integration of auditory and visual features of familiar objects. Initially, we replicated previous findings by observing AV enhancement of early visual (N1) event related potentials (Giard and Peronnet, 1999; Molholm et al., 2002; Molholm, 2004). Subsequent source modeling of the electrical signals revealed early AV integration responses in AT and IPC, starting from 50 to 100 ms, and preceding integration responses in the pO cortex (200–250 ms). This pattern of temporally interleaved integration responses supported an interactive account of multisensory integration, where early top–down feedback from higher-order regions biases or changes the inputs processed in the sensory-driven regions (Simons and Spiers, 2003). Although beyond the scope of this paper, determining the precise onset of these multisensory integration effects would be worthwhile investigating in future studies. The early effects (i.e., in the range 50–100 ms) should be interpreted cautiously, as their nature and functional significance may vary widely, depending the onset time (e.g., 50 ms or 80 ms).

Critically, these integration responses in the AT and IPC were modulated by semantic congruency, with enhanced responses for congruent stimuli. These early semantic effects suggested that AV integration responses in these regions differentially modulated integration in lower-level regions, as a function of the meaningfulness of the AV stimuli. Our findings agree with previous studies, which have reported semantic effects in similar regions, but have not investigated the time-course of their involvement in AV integration (Taylor et al., 2006; Hein et al., 2007). Unlike the present findings, stronger responses for incongruent than congruent trials have been reported in the IPC (Hein et al., 2007). This discrepancy may be explained by the different methodologies (fMRI vs. EEG), which do not yield directly comparable neural measures. Another important difference is the experimental task. The semantic congruency decision used here may drive stronger responses to stimuli that were felicitous with respect to the task description, than the passive viewing task used by Hein et al. (2007). The effect we observe is consistent with a context-specific role of the IPC, in particular to do with assessment of an object's meaning based on behavioral outcome (Miller and Cohen, 2001).

The early involvement of the AT region may be part of the mechanism for the rapid integration of stimuli from the auditory and visual modalities, and may underpin the enhanced behavioral performance reported for semantically congruent AV stimuli (Doehrmann and Naumer, 2008), including faster reaction times (Molholm, 2004) and improved target detection (Molholm, 2004). The AMTC has been found to integrate object features from different sensory modalities (Murray and Richmond, 2001; Taylor et al., 2006) and is modulated by AV semantic congruency (Taylor et al., 2006). In addition, the signal strength in the rhinal region has been found to correlate with the degree of an object's familiarity (Ranganath et al., 2004), and the perirhinal cortex has been implicated in familiarity-based object recognition (Aggleton and Brown, 1999). The rapid integration of AV properties in the AT region may involve simultaneous referencing of the sensory-specific representations in lower-level regions. When the stimuli are congruent, or can be integrated into a coherent, familiar object representation, a wide network of associations, strengthened through repeated exposures to the familiar object, may be activated to support the emergence of the AV object representation.

### Conflict of interest statement

The authors declare that the research was conducted in the absence of any commercial or financial relationships that could be construed as a potential conflict of interest.
